# An automatic nuclei segmentation method based on deep convolutional neural networks for histopathology images

**DOI:** 10.1186/s42490-019-0026-8

**Published:** 2019-10-17

**Authors:** Hwejin Jung, Bilal Lodhi, Jaewoo Kang

**Affiliations:** 1 Department of Computer Science and Engineering, Korea University, Seoul, Republic of Korea; 2 Interdisciplinary Graduate Program in Bioinformatics, Korea University, Seoul, Republic of Korea

**Keywords:** Nuclei segmentation, Deep learning, Histopathology, Image analysis, Convolutional neural network

## Abstract

**Background:**

Since nuclei segmentation in histopathology images can provide key information for identifying the presence or stage of a disease, the images need to be assessed carefully. However, color variation in histopathology images, and various structures of nuclei are two major obstacles in accurately segmenting and analyzing histopathology images. Several machine learning methods heavily rely on hand-crafted features which have limitations due to manual thresholding.

**Results:**

To obtain robust results, deep learning based methods have been proposed. Deep convolutional neural networks (DCNN) used for automatically extracting features from raw image data have been proven to achieve great performance. Inspired by such achievements, we propose a nuclei segmentation method based on DCNNs. To normalize the color of histopathology images, we use a deep convolutional Gaussian mixture color normalization model which is able to cluster pixels while considering the structures of nuclei. To segment nuclei, we use Mask R-CNN which achieves state-of-the-art object segmentation performance in the field of computer vision. In addition, we perform multiple inference as a post-processing step to boost segmentation performance. We evaluate our segmentation method on two different datasets. The first dataset consists of histopathology images of various organ while the other consists histopathology images of the same organ. Performance of our segmentation method is measured in various experimental setups at the object-level and the pixel-level. In addition, we compare the performance of our method with that of existing state-of-the-art methods. The experimental results show that our nuclei segmentation method outperforms the existing methods.

**Conclusions:**

We propose a nuclei segmentation method based on DCNNs for histopathology images. The proposed method which uses Mask R-CNN with color normalization and multiple inference post-processing provides robust nuclei segmentation results. Our method also can facilitate downstream nuclei morphological analyses as it provides high-quality features extracted from histopathology images.

## Background

Histopathology images are carefully and frequently assessed by pathologists to identify the presence and stage of a disease. However, conventional methods that rely on human assessment have limitations. First, when capturing and examining subtle visual features in complex histopathology images, the observations of human pathologists can vary for every examination. This can cause pathologists to disagree with each other even when assessing the same image.

In addition, as the number of pathologists decreases while the number of biopsy tests continues to increase, the workload of pathologists has been growing [1]. These problems can be alleviated by adopting deep learning and computer vision techniques. They can be used for improving accuracy, predicting the same results, and reducing the assessment time.

Conventional histopathology assessment is starting to leverage the power of deep learning to enhance diagnostic precision and is rapidly shifting towards computa- tional histopathology. Computational histopathology can be used for segmenting regions of interest, counting normal or cancer cells, recognizing tissue structures, classifying cancers, grading cancers, predicting the prognosis of cancer patients, and so on. Among these computational histopathology applications, we focus on nuclei segmentation in histopathology images.

Nuclei segmentation in histopathology images is challenging even for human pathologists for two main reasons. The first reason is the color variation in histopathology images. The H&E stain is one of the main stains used in histopathology. Hematoxylin stains nuclei while eosin stains other tissue structures; the background is not stained. However, staining protocols adopted by pathologists and the intensity of the stain can vary due to individual preferences or various organ types. Second, the differences in morphological structure can also be an obstacle in segmenting nuclei in histopathology images. As cells in different organs tend to have different morphological structures, the differences in the shape of individual cells should also be considered.

Several methods have been proposed to segment nuclei in histopathology images including the method by Otsu [2], the watershed method [3], K-mean clustering [4], Grab Cut [5], and so on. Furthermore, filtering based methods have been proposed to utilize the features of nuclei [6–8]. However, all of the above methods have the same major weaknesses. They are all extremely sensitive to parameter settings and are effective for only one or a few specific types of morphological nuclei structures. Since stains and morphological structures of nuclei can vary significantly, it is difficult to develop a generalized solution that can be applied to all histopathology images.

In recent years, machine learning based segmentation methods have been widely used due to their high performance. During the learning process, machine learning models have to be trained on the features of nuclei. Therefore, the features of nuclei need to be manually crafted and extracted. For example, features such as shape, color variance, color texture, blue ratio, color histograms, Laplacian of Gaussian response, geometric gradients, and other diverse features are extracted from histopathology images. Finally, these hand-crafted features are used for machine learning based methods to classify and distinguish nuclei from the background [9–12]. However, these methods are limited by their tedious and time consuming feature engineering.

Deep learning models that automatically extract features from raw data can alleviate these problems. Moreover, as deep learning models are robust due to their reliable performance in computer vision tasks such as object classification, detection, and segmentation, they are also shifting the paradigm of nuclei segmentation [13–17]. Xing et al. proposed a nucleus segmentation method that uses an iterative region merging algorithm and a deep learning model to initialize contours. Their nucleus segmentation method performs bottom-up shape deformation and top-down shape inference, and achieves good results [13].

Several studies have used a fully convolutional neural network (FCN) [18], which is a popular convolutional neural network (CNN) architecture, for object segmentation tasks. FCN is a CNN in which fully connected layers are replaced with convolutional layers. FCNs achieve high performance in various segmentation tasks in the computer vision field and nuclei segmentation [19, 20].

In addition, U-Net [21], which is based on FCN, has a sophisticated architecture with skip connections, and is used to segment nuclei in histopathology images. Cui et al. normalized colors of input images and trained a U-Net for segmentation [22]. Ronneberger et al. proposed a CNN based segmentation method, in which an FCN is used to produce three-class segmentation results (inside of nuclei, outside of nuclei, and boundary of nuclei) [21].

In the computer vision domain, state-of-the-art segmentation performance has been achieved by Mask R-CNN. Due to its outstanding object segmentation performance, Mask R-CNN has also been used for nuclei segmentation in microscopy images and achieved encouraging results [23]. However, there is still room for improvement in histopathology image segmentation. Thus, we apply Mask R-CNN as well as color normalization and multiple inference to segment nuclei in H&E stained histopathology images.

The main contributions of our study are listed below.

- We apply Mask R-CNN which is a state-of-the-art segmentation framework based on deep convolutional neural network to perform the nuclei segmentation task.
- We use the U-Net based deep convolutional Gaussian mixture color normalization model (DCGMM) to alleviate the large color variation in histopathology images.
- We use multiple inference for post-processing to improve the segmentation performance.
- We evaluate our nuclei segmentation method on two datasets which consist of histopathology images of various organs and histopathology images of the same organ, respectively. Our method achieves state-of-the-art performance on both datasets.

## Materials and methods

### Overview

**Fig. 1:**
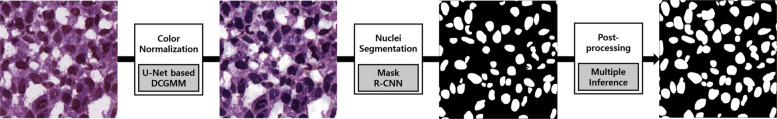
Workflow of our nuclei segmentation method

We use convolutional neural networks at various points to flexibly deal with difficulties of the nuclei segmentation task. Figure 1 shows the flow chart of our nuclei segmentation method. Our method includes the following four major steps: pre-processing, color normalization, nuclei segmentation, and post-processing. The details of each step are provided below. The codes for our nuclei segmentation method are available at the GitHub repository (https://github.com/hwejin23/histopathology_segmentation)

### Pre-processing

In the general computer vision field, an extremely large amount of data is required for training deep learning models. An insufficient amount of data may lead to the model overfitting the training data, which may result in poor testing performance. However, the datasets that we use for training and evaluation contained a very small number of histopathology images. Therefore, we apply several augmentation methods to increase the amount of data. Each image in the training set is randomly cropped, rotated (90^∘^,180^∘^, and 270^∘^), horizontally flipped, and vertically flipped. Therefore, we use training images which are enlarged by 1400 times.

### Color Normalization

**Fig. 2:**
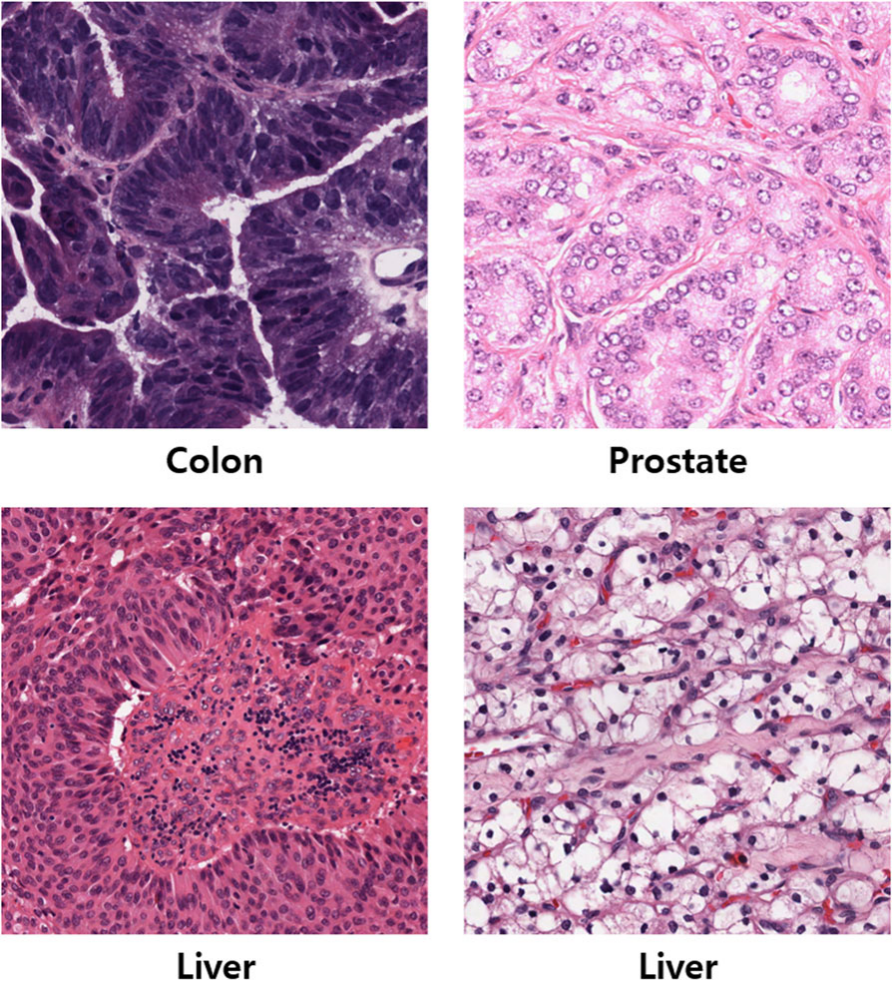
Different histopathology images with large color variations. The type of organ is indicted below each image

Color normalization is necessary due to the color variation in histopathology images. Figure 2 shows examples of histopathology images. As the examples show, there is a large color variation in the histopathology images. In the first row, the colon image seems to be over-stained while the prostate image can be considered under-stained. The two images in the second row are of the same organ (liver). It can be observed that the stained images differ even though the images are of the same organ. Moreover, the difference in color variation is more obvious when comparing all the images at once. Therefore, using color normalized image can improve segmentation performance. We use the deep convolutional Gaussian mixture color normalization model (DCGMM) [24] to reduce the color variation in histopathology images.

Several studies have devoted their efforts to developing robust color normalization methods for H&E stained histopathology images. Intensity thresholding [25], histogram normalization [26], stain separation [27], color deconvolution [28], and combining spatial information with color information [29] are representative normalization methods. The previously proposed DCGMM obtains state-of-the-art color normalization performance on H&E stained histopathology images with the large stain variations [24].

Conventional color normalization Gaussian mixture models have limitations since they cluster pixels based only on color attributes, without considering the spatial information or presence of an object. AF. G. Zanjani et al. applied a convolutional neural network (CNN) to a conventional Gaussian mixture model (GMM) for color normalization. DCGMM addresses limitations by fitting a Gaussian mixture model (GMM) with exploiting a CNN that helps capture the features of objects and their background.

The original DCGMM uses a naive CNN which consists of stacked convolutional layers [24]. DCGMM calculates the Gaussian distribution of object of each class based on segmentation results. Therefore, we replaced a naive CNN with a U-Net [21] which uses skip connections between layers and is known to be highly effective in medical image segmentation. The U-Net architecture that we use in this study is illustrated in Table 1.

**Table 1:** U-Net architecture used for the DCGMM in our study

Layer	Details		Layer	Details
Input			Output	
*↓*			*↑*	
*↓*			conv9_3	1x1x32; ReLU
*↓*			conv9_2	3x3x64; ReLU
conv1_1	3x3x32; ReLU		conv9_1	3x3x64; ReLU
conv1_2	3x3x32; ReLU	→	concat4	concatenate upsample4 with conv1_2
*↓*			*↑*	
pool1	2x2 max pool stride 2		*↑*	
*↓*			upsample4	2x2 upsample of conv8
conv2_1	3x3x64; ReLU		conv8	3x3x32; ReLU
conv2_2	3x3x64;ReLU	→	concat3	concatenate upsample3 with conv2_2
*↓*			*↑*	
pool2	2x2 max pool stride 2		*↑*	
*↓*			upsample3	2x2 upsample of conv7
conv3_1	3x3x128; ReLU		conv7	3x3x64; ReLU
conv3_2	1x1x128; ReLU		*↑*	
conv3_3	1x1x128; ReLU	→	concat2	concatenate upsample2 with conv3_3
*↓*			*↑*	
pool3	2x2 max pool stride 2		*↑*	
conv4_1	3x3x256; ReLU		upsample2	2x2 upsample of conv6
conv4_2	3x3x256; ReLU		conv6	3x3x128; ReLU
conv4_3	1x1x256;ReLU	→	concat1	concatenate upsample1 with conv4_3
*↓*			*↑*	
pool4	2x2 max pool stride 2		*↑*	
conv5_1	3x3x256; ReLU		upsample1	2x2 upsample of conv5_3
conv5_2	3x3x256; ReLU		*↑*	
conv5_3	1x1x256; ReLU	→	*↑*	

DCGMM is trained on a pixel-color distribution of the following tissue types: the nuclei, the surrounding tissues, and the background. DCGMM is optimized using a log-likelihood loss function and the gradient descent algorithm instead of the iterative expectation-maximization (EM) algorithm. In other words, the E-step of the EM algorithm is replaced by a CNN. The parameters *μ* and $\sum $ of multivariate Gaussian distributions of an input image are calculated similarly to the way they are calculated in the M-step of the EM algorithm.

In other words, training images are given to the U-Net based DCGMM and pixels are classified into one of the following groups: nuclei, surrounding tissues, and the background. The DCGMM calculates the distribution of clustered pixels. By an unsupervised method, the DCGMM is trained for 100000 iterations. The Adam optimizer [30] with a learning rate of 0.0001, beta1 of 0.9, beta2 of 0.999 and epsilon of 1e-0.8 are used for optimization.

Color normalization can be performed by adjusting the Gaussian distributions of input images using the Gaussian distributions of a template image. The template image and input images are inputted to the fully trained DCGMM and the parameters of the Gaussian distributions of the template image and input images are estimated. Then, the DCGMM calculates the Gaussian distribution of the input images using the Gaussian distribution of the template image. In our method, color normalization is applied to all the augmented histopathology images before training and testing Mask R-CNN, which is explained in the next section.

### Nuclei Segmentation

Mask R-CNN [31] is a state-of-the-art object segmentation framework that can identify not only the location of any object but also its segmented mask. Mask R-CNN extends the object detection model Faster R-CNN [32] by adding a third branch for predicting segmentation masks to the existing branches for classification and bounding box regression. Mask R-CNN is a two-stage framework. In the first stage, it scans an input image and finds areas that may contain an object using a Region Proposal Network (RPN). It predicts the classes of proposed areas, refines the bounding box, and generates masks for an object at the pixel level in the next stage based on the proposed areas from the first stage.

**Fig. 3:**
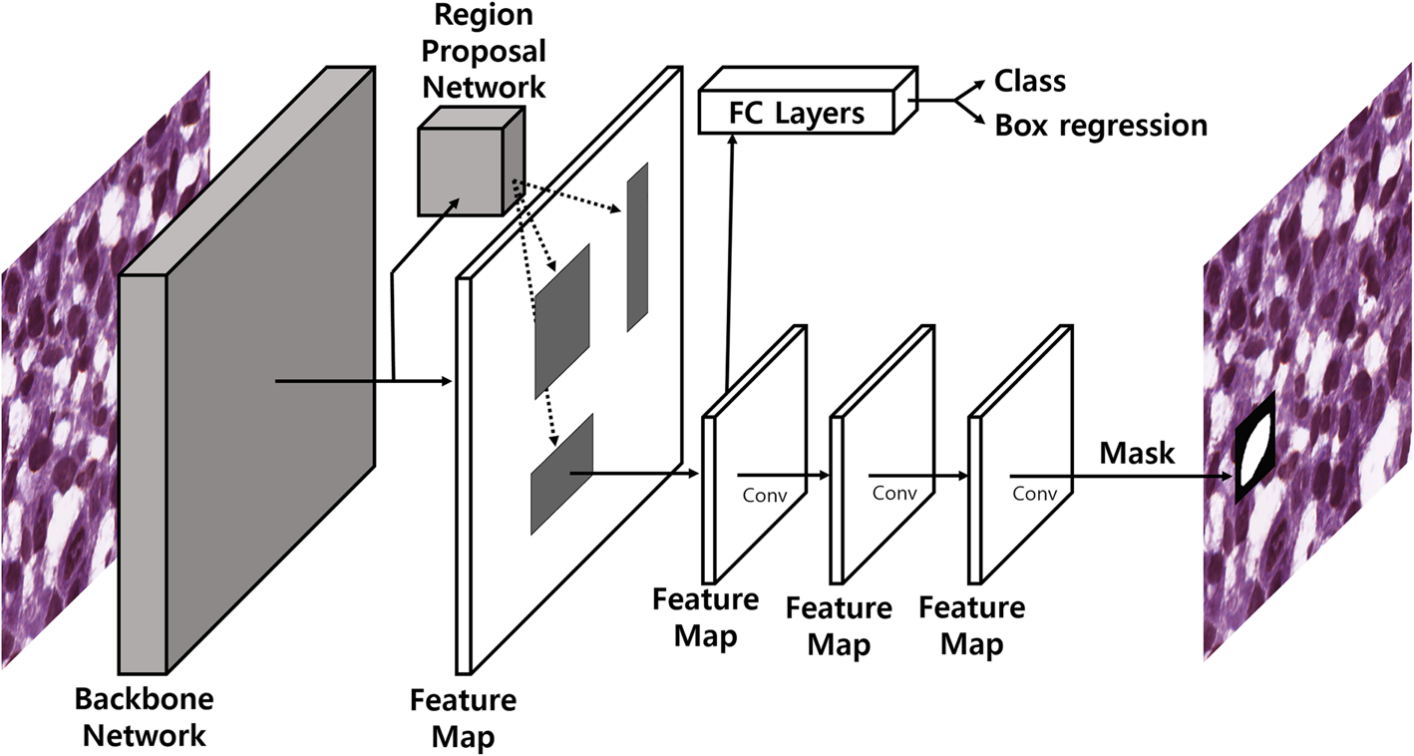
The overall network architecture of Mask R-CNN

Mask R-CNN framework has the following components: backbone network, region proposal network, object classifying module, bounding box regression module, and mask segmentation module. Figure 3 shows the overall architecture of Mask R-CNN. The backbone network is a standard convolutional neural network (CNN) that extracts features. Each input image is converted to a feature map by the backbone network and the feature map is used as the input for the following step. The Region Proposal Network (RPN) scans entire images to detect candidate areas that may contain objects. Instead of directly scanning an image, the RPN scans a feature map which is the output of the backbone network. The candidate areas distributed in the image are called "anchor boxes" and individually assessed. There are anchor boxes with different sizes and aspect ratios. Some anchor boxes can cover almost an entire image. The RPN has a confidence score for each anchor box. The confidence score of the anchor box indicates whether a given anchor box belongs to the background or foreground. A high classification score indicates that an anchor box likely contains part of an object. Since anchor boxes might not contain the entire object, the RPN refines the anchor boxes so that they can better fit the object, which is known as bounding box refinement.

For each anchor box containing an object, the object classification module and the bounding box regression module are applied. Unlike the RPN which predicts classes that are background and foreground, the object classification module is able to classify objects into specific classes including the background class. It classifies a given object into *n*+1 classes where *n* is the number of classes and 1 denotes the background class. The mask network is the main feature of Mask R-CNN. Although it is similar to the bounding box refinement process in RPN, Mask R-CNN performs a more detailed refinement of the location of the box. Finally, Mask R-CNN takes the foreground regions selected by the object classification module and generates masks for them.

Although we follow the general implementation of Mask R-CNN framework stated in the original paper [31]. For the backbone network, we employ a feature pyramid network (FPN) [33]. FPN consists of a bottom-up pathway, a top-bottom pathway, and lateral connections. A bottom-up pathway can be any convolutional network that extracts features from raw images. A top-bottom pathway sequentially generates same size of feature maps that correspond to feature maps generated by the bottom-up pathway.

Corresponding feature maps from the bottom-up pathway are added to the feature maps of the top-bottom pathway by the lateral connections. FPN outperforms other single convolutional networks mostly because it maintains semantically strong features at various resolution scales through its bottom-up pathway, top-bottom pathway and lateral connections. Among the various FPN architectures, we employ the FPN based on the ResNet-101 architecture. The weights of RPN based on ResNet-101 are pretrained on the ImageNet dataset. For anchors, we use the aspect ratios of 1:1, 1:2, and 2:1, and five scales with box areas of 8^2^,16^2^,32^2^,64^2^, and 128^2^. While the original Mask R-CNN used 5 scales with box areas starting from 128^2^, which is suitable for the COCO dataset, we modify the anchor sizes since nuclei are much smaller than the objects in the COCO dataset. We obtain segmentation results of Mask R-CNN on the top 1000 candidates to detect a large number of nuclei. A stochastic gradient descent (SGD) optimizer [30] with a learning rate of 0.001 and a learning momentum of 0.9 is used. In this study, DCGMM and Mask R-CNN were separately trained. We conduct all the experiments on a single machine with the following configuration: Intel(R) Core(TM) i7-6700 3.30GHz CPU with NVIDIA GeForce GTX 1070 Ti 8GB GPU and 48GB RAM.

### Post-processing

After training Mask R-CNN on the training set, we apply multiple inference to improve the segmentation results of our method. We augment each histopathology image in the test set by rotating (90^∘^,180^∘^, and 270^∘^), flipping horizontally, flipping vertically, and flipping horizontally and vertically. Augmentation methods that can change the size of an image are not applied. A total of 7 augmented images including the original image are generated and used as the input for multiple inference. After comparing one nucleus of the original image with all the nuclei of 7 augmented images, we selected nuclei with intersection over union (IoU) values greater than 0.2. For the segmentation results of the nuclei, majority voting at the pixel level is performed on the nuclei set we made. Pixels that have a score higher than 50% are selected as final segmented pixels. When we conduct inference, we use cropped images and restore them to original shaped images.

## Experiment and Results

### Datasets

We evaluate the performance of our nuclei segmentation method on two publicly available datasets. Both datasets consist of histopathology images and their corresponding ground-truth segmentation annotations.

The first dataset is the multiple organ H&E stained histopathology image dataset (MOSID) [20]. It contains a total of 30 images and the spatial size of each image is 1000×1000. Histopathology images of the following seven organs were collected: breast, kidney, liver, prostate, bladder, colon, and stomach. We divide the dataset into a training set and test set as shown in Table 2. Histopathology images of the bladder, colon, and stomach are included in only the test set.

**Table 2:** Composition of the multiple organ H&E stained histopathology image dataset (MOSID) which is divided into training and test sets

Data	Stained Images							
Division	Total	Breast	Kidney	Liver	Prostate	Bladder	Colon	Stomach
Training set	16	4	4	4	4	-	-	-
Test set	14	2	2	2	2	2	2	2
Total	30	6	6	6	6	2	2	2

Since the image size of 1000×1000 is too large for training our model, we set the input image size to 500×500 for the dataset that contains images of spatial size 1000×1000. Histopathology images for training are randomly cropped to the size of 500 × 500 as explained in the “Pre-processing” section and histopathology images for testing are divided into 9 overlapping sections. In other words, each section of a 1000×1000 sized histopathology image is cropped at the points of (0, 0), (0, 500), (500, 0), (500, 500), (250, 0), (0, 250), (250, 500), (500, 250), and (250, 250). When dividing an original image, the edges of nuclei may be cut off. To avoid this, we use overlapping sections. The 512×512 input size of the other dataset is small enough for training the model; no cropping or dividing is applied and histopathology images are used in their original form. After the data augmentation for training, around 1000 augmented images from MOSID are used.

The second dataset is the breast cancer histopathology image dataset (BNS) [19]. It consists of 33 H&E stained histopathology images and the spatial size of each image is 512×512. All the images are images of the breast. The images are collected from 7 breast cancer patients. While MOSID is divided into the training and test sets based on organs, BNS is divided based on patients. After the data augmentation for training, around 300–500 augmented images from BNS are used.

In addition to the datasets (MOSID and BNS) used for evaluating our nuclei segmentation method, an extra dataset is used for training the DCGMM with the U-Net. Among several datasets provided by the Tumor Proliferation Assessment Challenge 2016 (TUPAC)[34] organizers, we chose the auxiliary dataset which consists of images from three pathology centers and 73 breast cancer cases, without annotations for segmentation.

### Results

#### Evaluation metrics

To evaluate the performance of our nuclei segmentation method, we use two different evaluation metrics: object-level metric and pixel-level metric. F1 score is used as the representative evaluation object-level metric. F1 score is defined as (1) where TP is true positive, FP is false positive, and FN is false negative. Since F1 score is the harmonic average of the precision, which is defined as (2), and recall, which is defined as (3), F1 score is an ideal metric for evaluating both precision and recall at the same time.

1 $$ F1\:score = \frac{2TP}{2TP + FP + FN}   $$

2 $$ precision = \frac{TP}{TP + FP}   $$

3 $$ recall = \frac{TP}{TP + FN}   $$

One of the well known pixel-level metrics is Dice’s coefficient (DC) which is defined as (4) where X is the segmentation result and Y is its corresponding ground truth segmentation. Since this metric compares pixels with pixels, it can be used to evaluate the quality of the segmentation. Average Dice’s coefficient (ADC) can be calculated by averaging all the DC values. In addition Dice’s coefficient is also used for the criterion of F1 score that determines true positives, false positives and false negatives. Objects exceeding Dice’s coefficient value of 0.2 with the corresponding ground truth are determined as true positives.

4 $$ D(X,Y) = 2\frac{\vert X \cap Y \vert }{\vert X \vert + \vert Y \vert}   $$

ADC is limited in evaluating the pixel-level segmentation performance. It is biased towards correctly predicted results (true positives). In other words, false positive pixels and false negative pixels are completely ignored when assessing the segmentation quality. As calculating the number of false positives and false negatives is also important for evaluating the segmentation quality, we use the aggregated Jaccard index (AJI) which was proposed by Neeraj Kumar et al. [20]. Algorithm 1 is used to compute AJI.

AJI computes the number of intersection pixels and the number of union pixels between all ground truth pixels and segmented nuclei pixels. As AJI considers the number of false positive pixels and false negative pixels, it lowers the value of the results based on the errors.


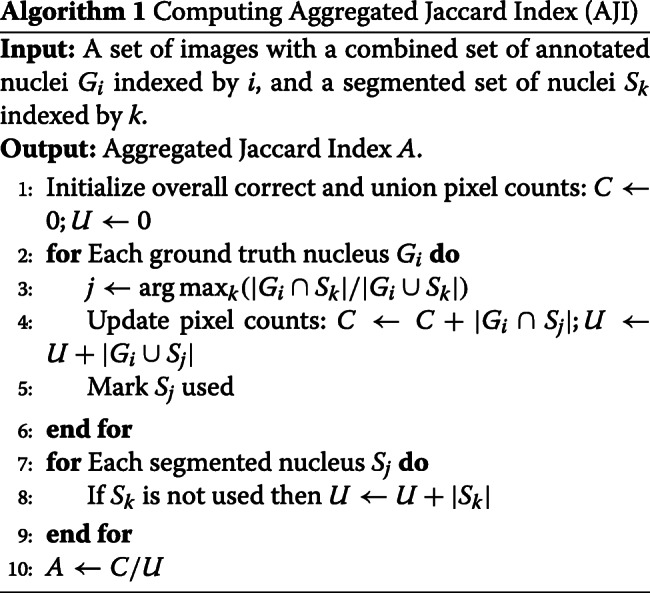


In our study, precision, recall, F1 score, Dice’s coefficient, and AJI are used as evaluation metrics for assessing the segmentation performance.

#### Experimental setups

We evaluate the performance of our nuclei segmentation method using different experimental setups. NucSeg refers to the experimental setup that uses Mask R-CNN, color normalization, and multiple inference. NucSeg-P denotes the experimental setup that does not use post-processing (multiple inference). NucSeg-N represents the experimental setup that uses post-processing but does not use color normalization. NucSeg-NP denotes the experimental setup which uses only Mask R-CNN. All the experimental setups are summarized in Table 3.

**Table 3:** Details of the experimental setups

	Color Normalization (DCGMM)	Nuclei Segmentation (Mask R-CNN)	Post processing (Multiple-Inference)
NucSeg	O	O	O
NucSeg-P	O	O	X
NucSeg-N	X	O	O
NucSeg-NP	X	O	X

#### Experiment 1 - MOSID

**Fig. 4:**
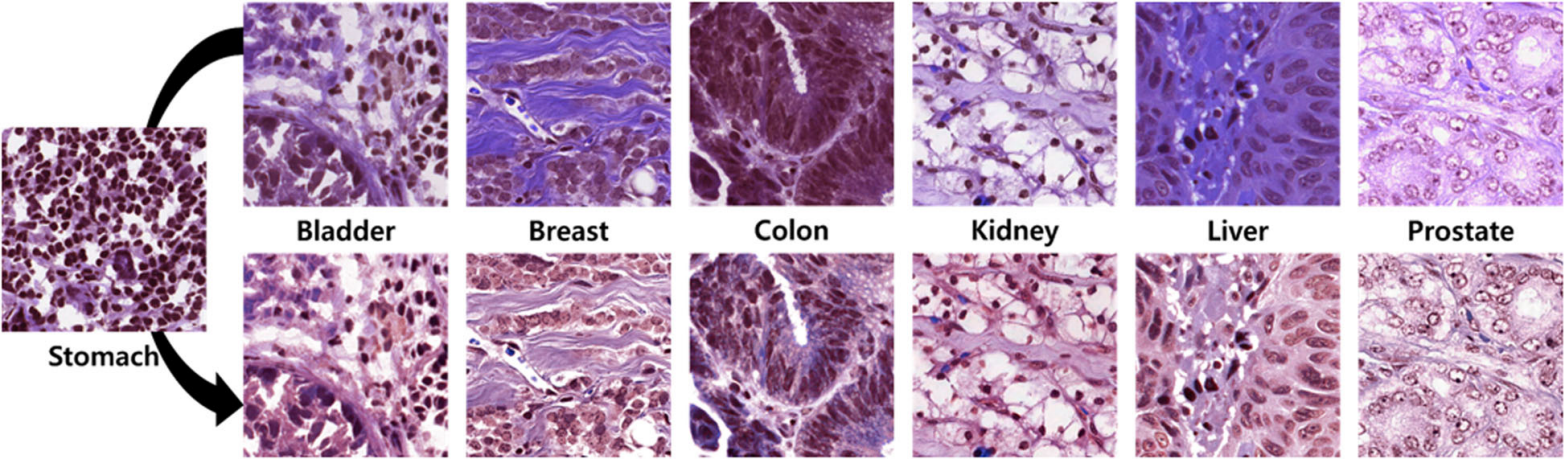
Top row shows original images of MOSID. Bottom row shows the same images after color normalization

Before the quantitative analysis, a qualitative analysis was performed. In Fig. 4, 6 histopathology images of different organs are normalized using histopathology images of stomach. For MOSID, we fix the input size of our U-Net based DCGMMs to 500×500. The histopathology images in MOSID show a relatively large color variation. The high color variation in MOSID images is due to the difference of organs. The images of MOSID after color normalization are clearer.

For a fair performance comparison, we measure the performance of our segmentation method and that of baseline segmentation methods on MOSID in the same way. The authors of the baseline methods randomly generated 5 different training sets and their corresponding test sets. They used the training and test sets to measure performance of their segmentation methods. Like the baseline authors, we also generate training and test sets randomly. Both the training and test sets are used to measure the overall performance of our method. In addition, We repeat our experiment 10 times and selected the different training and test sets each time. Table 4 shows the average and standard deviation of our results for the performance comparison with the baseline methods.

**Table 4:** Performance of several nuclei segmentation methods on the multiple organ H&E stained histopathology image dataset (MOSID)

Methods	Precision	Recall	F1-Score	ADC	AJI
CP [35]	N/A	N/A	0.405	0.597	0.123
Fiji [36]	N/A	N/A	0.665	0.649	0.273
CNN2 [13]	N/A	N/A	0.754	0.693	0.348
CNN3 [20]	N/A	N/A	0.827	0.762	0.508
NB [22]	0.836	**0.894**	0.852	0.809	N/A
**NucSeg**	**0.913 ±0*.*003**	0.821 ±0.004	**0.861 ±0*.*001**	**0.812 ±0*.*001**	**0.669 ±0*.*001**
**NucSeg-N**	0.897 ±0.004	0.813 ±0.004	0.849 ±0.002	0.805 ±0.002	0.649 ±0.004
**NucSeg-P**	0.909 ±0.002	0.777 ±0.004	0.835 ±0.002	0.809 ±0.001	0.664 ±0.002
**NucSeg-NP**	0.899 ±0.004	0.777 ±0.005	0.828 ±0.002	0.701 ±0.002	0.647 ±0.005

Performance obtained in NucSeg-P which uses color normalization is higher than that obtained in NucSeg-NP which only uses Mask R-CNN. Also, the performance obtained in NucSeg which uses both color normalization and multiple inference post-processing is higher than that obtained in NucSeg-N which uses only post-processing. These results demonstrate that color normalization helps properly train Mask R-CNN. In addition, the performance in NucSeg-N is higher than that in NucSeg-NP, and the performance in NucSeg is higher than that in NucSeg-P. Both results demonstrate that post-processing is beneficial. Comparing between color normalization and multiple inference post-processing, it appears that post-processing has more impact on the performance improvement as NucSeg-N slightly outperforms NucSeg-P. When both color normalization and multiple inference (NucSeg) are applied, all the performance scores of the metrics (precision, recall, F1 score, AJI, and Dice’s coefficient) increase, and the performance of our nuclei segmentation method improves.

Our nuclei segmentation method outperforms existing methods. Even in NucSeg-NP, which is the most basic setup, our method outperforms most of the other existing methods. The results of our method are much better than the results of CP [35] and Fiji [36], both of which are based on feature engineering. In addition, all of our experiments show that our method achieves better results than CNN2 [13] and CNN3 [20], both of which use shallow convolutional neural networks. As the baseline studies did not provide the precision and recall of their methods, a direct comparison of their precision and recall scores with ours is not possible. However, our method obtained a much higher F1-score, which demonstrates that our proposed method can achieve high performance in object level segmentation. Also, our segmentation method achieves higher AJI scores than existing methods. Higher AJI values represent a lower false positive to false negative ratio. Finally, since NB [22] used U-Net to segment nuclei in histopathology images which is dominant convolutional neural network architecture, comparing performance with NB [22] is more meaningful. In terms of all the evaluation metrics except recall, our segmentation method achieves higher scores on MOSID than scores achieved by NB [22] which is the state-of-the-art segmentation method.

#### Experiment 2 - BNS

**Fig. 5:**
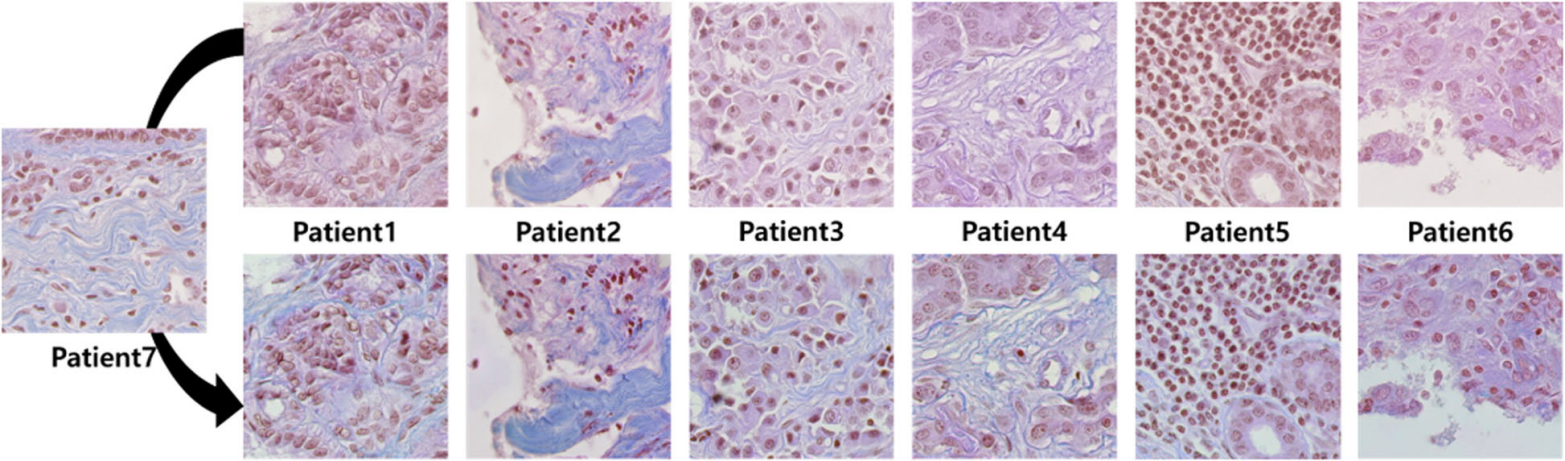
Top row shows original images of BNS. Bottom row shows the same images after color normalization

As shown in Fig 5, 6 histopathology images from 6 different patients are normalized using the other patient images. For BNS, we fix the input size of our U-Net based DCGMMs to 512×512. Comparing the first row of Fig. 4 with that of Fig. 5, the histopathology images in BNS have much less color variation than the histopathology images in MOSID. The changes of the MOSID and BNS images after color normalization are also noticeably different. Although there is a difference in degree of normalization, the color variation in the normalized images from BNS is smaller than in the original histopathology images.

To divide the BNS dataset into the training and test sets, we use leave-one-patient-out cross validation, which is the same evaluation strategy used in [19, 20]. As there are images from 7 patients, we train Mask R-CNN on images from 6 patients and test our nuclei segmentation method on images from the remaining patient. All the final metric scores tested on each patient are averaged and listed in Table 5.

**Table 5:** Performance comparison of several nuclei segmentation methods and our nuclei segmentation method evaluated on the breast cancer histopathology image dataset (BNS)

Methods	Precision	Recall	F1-Score	ADC	AJI
PANGNET [18]	0.814	0.655	0.676	N/A	N/A
FCN [18]	0.823	0.752	0.763	N/A	N/A
DeconvNet [37]	0.864	0.773	0.805	N/A	N/A
Ensemble [19]	0.741	0.900	0.802	N/A	N/A
NB [22]	**0.920**	0.784	0.840	0.830	N/A
**NucSeg**	0.907	**0.923**	**0.913**	0.835	0.686
**NucSeg-N**	0.910	0.910	0.909	**0.838**	**0.688**
**NucSeg-P**	0.893	0.886	0.887	0.810	0.654
**NucSeg-NP**	0.912	0.889	0.899	0.818	0.665

Multiple inference helps to boost the performance of our nuclei segmentation method. However, color normalization does not help to improve the segmentation performance on BNS because the color variation of the images in BNS is already small. Since the images in MOSID have a large color variation and images in BNS have a small color variation, color normalization played a major role in improving the segmentation performance on MOSID and played an insignificant role in enhancing the performance on BNS. In MOSID, all the images are of different organs and have a large color variation. However, BNS consists of histopathology images of the same organ. In other words, BNS has much less color variation than MODIS; thus, Mask R-CNN can be trained on BNS without difficulty.

As demonstrated, our nuclei segmentation method outperforms existing methods. There are several segmentation methods that perform the segmentation task on BNS. The overall results of our method and the segmentation methods based on deep convolutional neural network are shown in Table 5. As shown in Table 5, our segmentation method achieves state-of-the-art performance. Our method obtains better ADC scores and F1-scores than NB [22], the state-of-the-art segmentation method evaluated on BNS. This result shows that our model obtains better segmentation performance than NB.

## Discussion

**Fig. 6:**
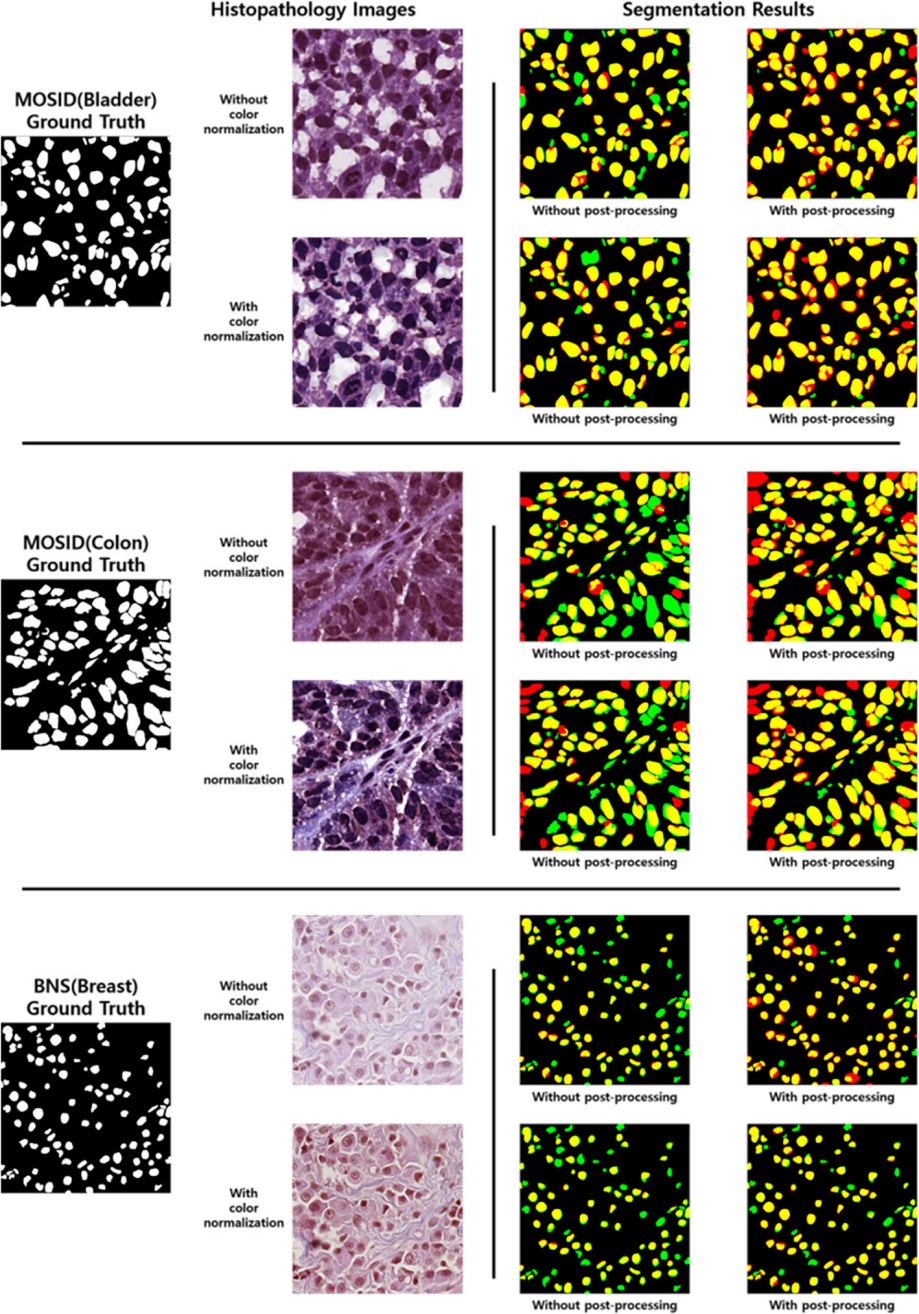
Several histopathology images of MOSID and BNS and their segmentation result images to which our segmentation method is applied. In the segmentation result images, the yellow areas denote true positive pixels, red areas denote false positive pixels, and green areas denote false negative pixels

Figure 6 shows several histopathology images and corresponding segmentation result images to which our segmentation method is applied. We use bladder and colon images from MOSID and a breast image from BNS. As shown, histopathology images become clearer after applying color normalization. In addition, segmentation result images of input images with and without color normalization are also presented in Fig. 6. Yellow areas denote true positive pixels, red areas denote false positive pixels, and green areas denote false negative pixels. In other words, green and red indicate segmentation errors.

For MOSID histopathology images, our method achieves the best performance on bladder images and the lowest performance on colon images. It is easier to distinguish nuclei from the background in bladder images than in colon images. In addition, we find inaccurate and missing annotations. First, some ground truth annotations of nuclei on the edges of histopathology images are missing. Second, the criteria for making ground truth annotations of nuclei for each histopathology image are different. These inaccurate annotations result in lower performance for some histopathology images.

Our segmentation method significantly outperforms existing methods on BNS. However, its performance on MOSID only slightly improves due to color normalization. Since BNS histopathology images have a small color variation, color normalization is not that helpful. For this reason, we find that color normalization is only effective when histopathology images have a large color variation.

Overall, more error areas are observed in the segmentation result images without post-processing. Also, more error areas are observed in the segmentation result images of histopathology images to which color normalization is not applied. As discussed in the “Results” subsection, Fig. 6 intuitively shows that the segmentation images with color normalization and post-processing are the best.

## Conclusion

In this paper, we proposed a method for nuclei segmentation in histopathology images. Mask R-CNN which obtains state-of-the-art performance on the nuclei segmentation task was used. Performance improvement due to the U-Net based deep convolutional Gaussian mixture color normalization model (DCGMM) showed that color normalization enhances performance on datasets containing histopathology images with large color variations. Furthermore, the multiple inference method for post-processing improved the segmentation performance on each test image. The performance comparison demonstrates that our nuclei segmentation method is more robust than the state-of-the-art segmentation methods.

## Data Availability

MOSID used for the current study is available in the repository at https://monuseg.grand-challenge.org/Data/. BNS used for the current study is available in the repository at https://peterjacknaylor.github.io/PeterJackNaylor.github.io/2017/01/15/Isbi/. TUPAC used for the current study is available in the repository at http://tupac.tue-image.nl/.

## Abbreviations

ADC: Average dice coefficient
AJI: Aggregated Jaccard index
BNS: Breast cancer histopathology image dataset
CNN: Convolutional neural network
DC: Dice coefficient
DCGMM: Deep convolutional Gaussian mixture color normalization
EM: Expectation maximization
FCN: Fully convolutional neural network
GMM: Gaussian mixture model
IoU: Intersection over union
MOSID: Multiple organ H&E stained histopathology image dataset
RPN: Region proposal network
SGD: Stochastic gradient descent
TUPAC: Tumor proliferation assessment challenge 2016
